# Patient preferences for models of care for fibromyalgia: A discrete choice experiment

**DOI:** 10.1371/journal.pone.0305030

**Published:** 2024-06-21

**Authors:** Patrícia Norwood, Marcus Beasley, Martin Stevens, Rosemary Hollick, Gary Macfarlane, Paul McNamee, for the PACFiND Study Investigators

**Affiliations:** 1 Health Economics Research Unit, School of Medicine, Medical Sciences and Nutrition University of Aberdeen, Aberdeen, United Kingdom; 2 Aberdeen Centre for Arthritis and Musculoskeletal Health (Epidemiology Group), School of Medicine, Medical Sciences and Nutrition, University of Aberdeen, Aberdeen, United Kingdom; University of Catanzaro: Universita degli Studi Magna Graecia di Catanzaro, ITALY

## Abstract

**Background:**

Fibromyalgia is a common reason for referral to a rheumatologist and is a centralised pain state with symptoms beginning in adolescence/early adulthood and manifests as pain throughout the body, fatigue and cognitive dysfunction. Whilst there is considerable evidence on effective treatments, diagnosis and management are complex. There is almost no evidence on how to organise health services to deliver recommended therapies. The aim of the current study was to understand patient preferences for different features of healthcare services for fibromyalgia.

**Methodology:**

We use the Discrete Choice Experiment Method (DCE), a choice-based survey that quantifies preferences for attributes of goods, services or policy interventions, to elicit preferences in relation to alternative models of care for people with fibromyalgia. In this study, attributes describe different models of care for fibromyalgia. We based attributes and levels on earlier phases of the PACFiND project and a literature review on fibromyalgia models of care. The final analysis sample consisted of 518 respondents who completed the survey in full.

**Results:**

The final analysis sample consisted of 518 respondents ((patients living in the UK, over 18 years old, with a diagnosis of fibromyalgia), who completed the survey in full. The model of care most preferred is one characterised by earlier diagnosis and ongoing management by a Rheumatologist, via Face-to-face or Phone/video call appointments, with a stronger preference for the latter mode of support. The most preferred treatment was Medication, followed by Physical Therapy, with the least preferred being Talking Therapy. Relative to a Waiting Time for treatment of 6 months, respondents would prefer a lower Waiting Time of 3 months and dislike waiting 12 months for treatment. Respondents showed willingness to receive Ongoing Help and Advice by a Nurse Practitioner or a GP, instead of a Specialist Rheumatologist, provided they were compensated by other changes in the model of care.

**Conclusion:**

This study has found that, although respondents express a preference for specialist care, provided by a Rheumatologist, they may be willing to trade-off this preference against other features within a model of care. This willingness to accept a different skill-mix (e.g., appointments with a GP or a Nurse Practitioner) has important implications for practice and policy, as this is a more feasible option in settings where the availability of specialist care is highly constrained.

## Introduction

Fibromyalgia is a common reason for referral to a rheumatologist [1] and is estimated to affect from around 1 in 50 to 1 in 20 people, depending on the criteria used for classification [2]. Estimates of population prevalence are highly variable; for example, the population prevalence of fibromyalgia in the UK was estimated to be around 5.4% in 2015 [2] but, depending on the classification criteria, it can be considerably higher in other countries, for example, in Saudi Arabia, where it has been estimated to be 13.4% [3]. It is a pain state of no clear pathophysiological mechanism, with symptoms usually beginning in adolescence/early adulthood, although they can start later in life as well, and manifest as pain throughout the body, fatigue and cognitive dysfunction. The condition has a large impact on quality of life, with psychological distress prominent in comparison to other pain conditions, [4]. Elevated risks of suicide and suicidal ideation have been reported [5] and increasing focus on treatments that aim to improve mental health, such as cognitive behavioural therapy, are now being evaluated in many health care settings globally [6], alongside established treatment such as medication and physical therapy (e.g. exercise).

Fibromyalgia can be difficult to diagnose as many symptoms are similar to those seen in other conditions. Some individuals can wait many years for a diagnosis, involving many general practice consultations and referral to a number to different specialists [7,8]. Whilst there is considerable evidence on effective treatments [9], most individuals with fibromyalgia are not receiving timely diagnosis, access to effective treatments or ongoing support with managing their condition. There is almost no evidence on how to organise health services to deliver recommended therapies and patients feel dissatisfied with current services believing that no-one is willing to take responsibility for their care [10].

We conducted a Discrete Choice Experiment (DCE), as one of the phases of a larger programme of work, with the aim of developing a new model of care for people with fibromyalgia symptoms—PAtient-centred Care for Fibromyalgia: New pathway Design (PACFIND). The DCE method is a flexible approach to estimate the value of different models of care or treatments in terms of perceived benefits to patients, having previously been used in patients with musculoskeletal conditions [11–18]. However, no research to date has been conducted with patients experiencing fibromyalgia.

The aim of the current study was to understand patient preferences for characteristics of healthcare services for fibromyalgia.

## Methods

### Designing the DCE

A DCE is a choice-based survey that quantifies preferences for attributes (or features) of goods, services or policy interventions. It assumes that any good or service (in this case models of care for fibromyalgia) can be described by its attributes and the measure (or ‘levels’) of these attributes. Each respondent faces a series of hypothetical scenarios (choice sets) composed of two or more alternatives [19]. In each choice set, respondents are asked to choose their preferred scenario. The principle underlying a DCE is that choices are made based on the features (or attributes) of different options. Therefore, a DCE enables researchers to gain insight into the relative importance of each attribute and the trade-offs between attributes [20].

In this study, attributes describe different models of care for fibromyalgia. We based attributes and levels on earlier phases of the PACFiND project, which reviewed the literature on fibromyalgia models of care and used surveys and qualitative interviews with health and social care professionals and patient experiences of using health care [7] to assess current models of delivery of care for patients diagnosed with fibromyalgia [10]. Analysis of that information, working alongside the project’s Patient and Public Involvement (PPI) partners, yielded six attributes, with associated levels, shown in Table 1.

**Table 1:** List of attributes and levels.

Attributes	Levels	Variable in equation
Time to diagnosis	1 year	TimeD
	2 years	
	3 years	
	5 years	
Who makes the diagnosis	GP	DiagGP
	Rheumatologist	DiagRheu
Type of treatment	Medication	TreatMed
	Physical Therapy	TreatPT
	Talking Therapy	TreatTT
Wating time for treatment	3 months	TimeT
	6 months	
	12 months	
Ongoing help and advice	Appointments with GP	HelpGP
	Appointments with Rheumatologist	HelpRheu
	Appointments with Nurse Practitioner	HelpNurse
	Peer Support	HelpPeer
How ongoing help and advice is provided	Face-to-face appointments	OngF2F
	Phone-call/video-call appointments	OngCall
	Text messaging	OngText

^a^ Time to diagnosis from the start of first symptoms.

^b^ Health care provider that makes the initial diagnosis.

^c^ Type of help and advice received after the diagnosis.

^d^ Patient would be prescribed a medicine, or a set of medicines or drugs, used to improve fibromyalgia symptoms.

^e^ The patient would undertake a programme of physical exercise designed by a trained exercise professional. The type of exercise and the amount of time would be adjusted to suit their own need.

^f^ The patient would undertake a training programme designed by a trained behavioural therapist. The programme would involve focusing on their current thoughts, beliefs and attitudes, how these affect feelings and behaviour, and learning coping skills to deal with problems.

^g^ Following diagnosis, the waiting time to receive help and advice from a health care provider.

^h^ Ongoing appointments to manage fibromyalgia.

^I^ How the ongoing help and advice would be provided.

Two of the attributes refer to patients’ preferences regarding how long it takes to be diagnosed (1 year, 2 years, 3 years, 5 years) and who would they prefer to be diagnosed by (GP or Rheumatologist). Preferences for treatment were described with attributes describing types of treatment (Medication, Physical Therapy, Talking Therapy) and Waiting times for Treatment (3 months, 6 months, 12 months). The remaining attributes described who provides Ongoing Help and Advice (Appointments with GP, Appointments with Rheumatologist, Appointments with Nurse Practitioner and Peer Support) as well as mode of provision of Ongoing Help and Advice (Face-to-face appointments, Phone-call/Video-call appointments, Text messaging).

Twelve choice sets were selected using Ngene design software (with one additional choice set to test for response consistency) with each choice set consisting of two alternative modes of care delivery. Patients were asked to compare the two alternative services and choose which one they would prefer. An example choice set is shown in S1 Fig.

In the last section of the survey participants were asked to answer questions on socio-demographic characteristics. These observable characteristics were used to characterise preference heterogeneity.

The survey was developed *de-novo*, so no permissions were required.

### Recruitment and administering the DCE questionnaire

An initial version of the survey was created, and nine fibromyalgia patients recruited through PACFiND’s Patient and Public Involvement (PPI) group were asked to complete the survey and conduct a Think-aloud exercise while doing it. A further three patients from the PPI group were asked to complete the survey and provide written feedback. A pilot survey was developed that addressed the feedback obtained from the Think-aloud exercise and the written comments, and a paper version was mailed to 75 people with fibromyalgia who participated in a previous phase of PACFiND and had agreed to be contacted again (41 returned completed surveys).

The final version of the questionnaire was administered as an online survey using Qualtrics software. Participants were recruited to the survey via links shared on social media, in newsletters, and on websites, of relevant charity and support groups, including Versus Arthritis and Fibromyalgia Action UK. Recruitment also took place through emailed invitations to the Patient-Partner Involvement group of the Epidemiology Group of the University of Aberdeen. Additionally, the survey was announced on the PACFiND project website (hosted by the University of Aberdeen), the PACFiND Facebook and Twitter feeds, and the Twitter feeds of the PACFiND investigators. Participants were asked, in the online survey, to confirm they consented to participate in the survey.

Data were collected between 10^th^ of January and 18^th^ of February 2022. Apart from the DCE questions, the questionnaire also included a section where participants were asked to rank the attributes of care and express their preference for each attribute through explicit questions, such as specifying their preferred healthcare provider for diagnosis (e.g., GP or Rheumatologist). Additionally, socio-demographic and diagnostic information including age, gender, year of diagnosis, employment status, education level, and household income were collected.

Full ethical approval for the survey was obtained from the University of Aberdeen’s School of Medicine, Medical Sciences and Nutrition Ethics Review Board (SERB/2021/10/2182).

### Data analysis

Analysis of the data was based on random utility theory. From the DCE questions respondents choose one scenario from two presented in each choice task. We assume that, in each choice task, respondents choose the alternative that would provide them with the highest utility. The link between observed choices and changes in the attributes is made possible by the random utility maximisation (RUM) framework [21].

Eq 1, below, was estimated using a mixed logit model:

$$V_{j}=\beta_{0}+\beta_{1}TimeD+\beta_{2}DiagGP+\beta_{3}DiagRheu+\beta_{4}TreatMed+\beta_{5}TreatPT+\beta_{6}TreatTT+\beta_{7}TimeT3+\beta_{8}TimeT3+\beta_{9}TimeT12+\beta_{10}HelpGP+\beta_{11}HelpRheu+\beta_{12}HelpNurse+\beta_{13}HelpPeer+\beta_{14}OngF2F+\beta_{15}OngCall+\beta_{16}OngText$$ (1)

The utility derived from the model of care for people with fibromyalgia is represented by V_j,_ which is characterised by different combinations of the attribute’s levels. β_0_ is the alternative specific constant and it indicates a general preference to choose Service A over Service B, everything else constant. A positive (negative) coefficient means respondents are less likely to choose service B (A) over Service A (B), everything else being equal.

The sign of the coefficients (β1 to β13) points to whether a change in the attribute level has a positive or negative impact on utility of fibromyalgia model of care. Effects coding, which allows for the estimation of all the levels of categorical variables, was used for all the variables except for Time to diagnosis (TimeD) which was coded as continuous. The coefficient of TimeD represents the effect on utility of a 1-year increase in the Waiting Time for diagnosis. The remaining coefficients represent the impact of the presence of that attribute level on the level of utility. For instance, β_2_ represents the additional utility of being diagnosed by a GP (relative to a Rheumatologist) while β_3_ represents the additional utility of being diagnosed by a Rheumatologist (relative to a GP).

The regression coefficients provide information on whether a change in an attribute’s level has a significant positive, significant negative, or no significant effect on a scenario’s utility. The trade-offs between any two attributes are represented by the ratio of these coefficients. For example, the trade-offs respondents make between the Time to Diagnosis (TimeD) and other attributes represents respondents’ value of a unit change in an attributes’ level. Calculating this trade-off between each attribute’s levels converts the regression coefficients into a meaningful and comparable metric (in this case, Waiting Time to Diagnosis).

From Eq 1, the Willingness-to-Wait (WTW) for a marginal change in an attribute can be estimated as the ratio of that attribute with TimeD. So, for example, the Willingness-to-Wait for a Diagnosis (WTWD) to receive Medication as the treatment of choice can be calculated as WTWD Medication = – (β_4_/ β_1_)

WTWD values were estimated for all statistically significant attributes.

## Results

A total of 957 people accessed the survey with 10 excluded because they did not have a diagnosis of fibromyalgia or were not UK residents. Only fully completed surveys were considered so the final analysis sample consisted of 518 respondents. Table 2 shows respondents characteristics.

**Table 2:** Characteristics of respondents to the DCE.

	n(%)
**Age**	Min = 21 Max = 83 Mean = 48
**Years since diagnosis**	Min = 1 Max = 36 Mean = 8
**Gender**	
Male	28 (5.4)
Female	482 (93.1)
Non-binary/Third gender	7 (1.4)
Prefer not to say	1 (0.2)
**Employment status**	
Part-time employment	85 (16.4)
Full-time employment	119 (23.0)
Self-employed	27 (5.2)
Unemployed Retired	94 (18.1) 61 (11.8)
Student	6 (1.2)
Other	126 (24.3)
**Highest level of Education completed**	
Secondary School	86 (16.6)
Vocational/Trade/College Qualification	128 (24.7)
Highers/A levels	81 (15.6)
University Qualification	223 (43.1)
**Household Income**	
Up to £10,000	98 (18.9)
£10,001-£20,000	138 (26.6)
£20,001-£30,000	114 (22.0)
£30,001-£40,000	58 (11.2)
£40,001-£50,000	48 (9.3)
£50,001 +	62 (12.0)

Respondent age was on average 48 years (range: 21 to 83yrs). The average time since being diagnosed with fibromyalgia was 8 years (range: 1 to 36yrs). The vast majority of respondents were female (93%), consistent with existing data on sex ratio of fibromyalgia diagnosis [22], rather than the prevalence of people meeting more recent criteria [2], e.g. in one study, fibromyalgia is diagnosed in women in a proportion of 9:1 [23]. Just under a quarter of respondents were in full-time employment (23%), 16.4% were in part-time employment, 18.1% were retired and 5.2% were unemployed. Almost a quarter of respondents chose “Other” as an option when questioned about their employment status. This was a free text option where 65% of respondents stated they were unable to work due to disability (around 16% of total respondents), and 16% had taken early retirement due to disability (around 3% of total respondents). In terms of the highest level of education attained, 16.6% had completed secondary school, 24.7% had vocational/trade/college qualifications, 15.6% had Higher/A levels and 43.1% had a university qualification. When asked to choose which category represented their household income from all sources (before tax and other deductions), 18.9% were under £10,000, 26.6% earned between £10,001 and £20,000, 22% between £20,001 and £30,000, 11.2% between £30,001 and £40,000, 9.3% between £40,001 and £50,000 and 12% reported more than £50,001.

Table 3 shows the regression results and WTWD for all respondents.

**Table 3:** Results of regression analysis and WTWD values (measured in months and years).

	Coefficient	P-value	95% Conf. Interval		WT Wait for a Diagnosis (years)	WT Wait for a Diagnosis (months)
asc	0.07	0.00	0.04	0.10	0.17	2.02
**Who makes diagnosis**						
*GP*	-0.22	0.00	-0.27	-0.18	-0.53	-6.39
Rheumatologist	0.22	0.00	0.18	0.27	0.53	6.39
**Type of treatment**						
*Talking Therapy*	-0.41	0.00	-0.50	-0.31	-0.97	-11.69
Physical Therapy	0.15	0.00	0.08	0.21	0.35	4.18
Medication	0.26	0.00	0.18	0.34	0.63	7.51
**Who provides ongoing help and advice**						
*Peer Support*	-0.36	0.00	-0.44	-0.28	-0.86	-10.32
Appointments with nurse practitioner	-0.02	0.56	-0.10	0.05	n.s.	n.s.
Appointments with Rheumatologist	0.43	0.00	0.34	0.52	1.03	12.36
Appointments with GP	-0.05	0.20	-0.12	0.03	n.s.	n.s.
**How ongoing help and advice is provided**						
*Text messaging*	-0.41	0.00	-0.47	-0.34	-0.97	-11.65
Phone call/video call appointments	0.26	0.00	0.20	0.32	0.62	7.47
Face-to-face appointments	0.15	0.00	0.09	0.20	0.35	4.18
**Time to diagnosis**	-0.42	0.00	-0.45	-0.39		
**Time to treatment**						
*3 months*	0.26	0.00	0.18	0.34	0.62	7.48
6 months	-0.08	0.31	-0.24	0.08	n.s.	n.s.
12 months	-0.18	0.00	-0.28	-0.08	-0.43	-5.12

Number of observations = 12,432.

Log likelihood = -3442.1878.

LR chi2(11) = 322.13.

Prob > chi2 = 0.000.

Time to diagnosis was negative and significant, indicating that time played an important part in respondents’ preferences, and they preferred to wait less time for a diagnosis. Respondents preferred being diagnosed by a Rheumatologist. They were willing to wait just over half a year longer (-6.4 months; P<0.001) to be diagnosed by a Rheumatologist rather than a GP. In terms of Type of Treatment, respondents showed a strong preference for not having Talking Therapy, with a willingness to wait for almost a full year longer for diagnosis (11.7 months; P<0.001) to avoid having Talking Therapy as the treatment of choice. Respondents showed a preference for Physical Therapy (WTWD -4.2 months; P<0.001) and Medication (WTWD -7.5 months; P<0.001). Regarding the provision of Ongoing Help and Advice, there was a preference for being seen by a Rheumatologist, with respondents willing to wait over a year longer (WTWD -12.4 months; P<0.001) for diagnosis if their Ongoing Care was provided by a specialist, relative to being seen by a Nurse Practitioner or a GP. Peer Support was not a preferred option (WTWD 10.3 months; P<0.001). When considering Time to Wait for Treatment, there was a preference for a wait of 3 months (WTWD -7.5 months; P<0.001), a wait of 6 months was not significant, and respondents were averse to wait for 12 months (WTWD 5.1 months; P<0.001).

In comparing the explicit preferences elicited from the questionnaire with the DCE findings, consistent agreement was observed for each attribute with an exception for the mode of provision for Ongoing Help and Advice. While the DCE questions highlighted a stronger preference for Phone/video call appointments for Ongoing Help and Advice, the explicit questions revealed a stronger inclination towards Face-to-face appointments among the respondents (WTWD -4.2 months for Face-to-face versus WTWD -7.5 months for Phone/video call).

Table 4 describes the least preferred and most preferred fibromyalgia models of care.

**Table 4:** WTWD (months) of worst and best models of care.

	Worst Model of care	Best Model of care
**Constant**	**2.02**	**2.02**
**Who makes diagnosis**	** **	
GP	**-6.39**	** **
Rheumatologist	** **	**6.39**
**Type of treatment**	** **	** **
Talking Therapy	**-11.69**	** **
Physical Therapy	** **	** **
Medication	** **	**7.51**
**Who provides ongoing help and advice**	** **	** **
Peer Support	**-10.32**	** **
Appointments with nurse practitioner	** **	** **
Appointments with Rheumatologist	** **	**12.36**
Appointments with GP	** **	** **
**How ongoing help and advice is provided**	** **	** **
Text messaging	**-11.65**	** **
Phone call/video call appointments	** **	**7.47**
Face-to-face appointments	** **	** **
**Time to diagnosis**	** **	** **
**Time to treatment**	** **	** **
3 months	** **	**7.48**
6 months	** **	** **
12 months	**-5.12**	** **
**TOTAL**	**-43.16**	**43.24**

A model of care where the respondent was diagnosed by a GP, offered Talking Therapy and Peer Support, Ongoing Help and Advice provided via Text Message, and a wait for the beginning of Talking Therapy and Peer Support of 12 months, would bring the least utility, with a total WTWD of -43.2 months, that is respondents would be willing to wait 43.16 months (around 3.5 years) to avoid having this model of care:

WTWD = 2_constant_ -6.4_DiagGP_− 11.7_TreatTT_ -10.3_HelpPeer_− 11.7_OngText_− 5.1_TimeT12_ = -43.2 months

The most preferred model would be one where both the diagnosis and the follow-up care were undertaken by a Rheumatologist, with ongoing Phone-call/video-call appointments, where the treatment would consist of Medication and would start within 3 months of diagnosis:

WTWD = 2_constant_ + 6.4_DiagRheu_ + 7.5_TreatMed_ + 12.4_HelpRheu_ + 7.5_OngCall_ + 7.5_TimeT3_ = +43.2 months

S2 Fig shows several trade-offs that respondents say that they are willing to make between different characteristics of the fibromyalgia care model and what impact it would have on their level of utility, as measured by total WTWD.

If the starting point was the least preferred model of care, respondents’ WTWD would improve by 12.8 months if, instead of being diagnosed by a GP, the diagnosis was made by a Rheumatologist. A plan of treatment that used Medication, as opposed to Talking Therapy, would also contribute to an increase in utility (19.2 months). If the follow-up care was done by a Rheumatologist, the utility would further increase by 21.2 months but if it was done by a GP, the level of utility would decrease by 10.9 months and lead to a negative level of utility (WTWD = -2.9). That drop in the level of utility could be compensated by Ongoing Help and Advice provided via Phone/video calls instead of Text Messages (increase in WTWD of 15.8 months) and a Waiting Time for Treatment of 3 months, rather than 12 months, which would increase the WTWD by 12.6 months, leading to a total WTWD of 25.6 months.

Findings from the likelihood ratio tests, used to test if preferences differed across subgroups, indicated that age was significantly associated with preferences (S3 Fig).

Looking at two groups split by mean age (20–48 years old and 49–83 years old), there were no significant differences relating to preferences for who makes the diagnosis and Time to Treatment. In terms of Type of Treatment, preferences for Physical Therapy and Talking Therapy were not statistically different from each other but the younger group showed a much stronger preference for Medication, where they were willing to wait 10.7 months for diagnosis to get Medication versus only 4.5 months in the older group. Preferences for who provides Ongoing Help and Advice were quite similar, with the exception that the older group preferred not to be followed up by a GP, but the younger group were indifferent to it. Regarding how Ongoing Help and Advice was provided, the younger group was significantly less averse to Text Messaging (WTWD of 8.3 months vs 14.5 months) and, while the older group positively valued having Face-to-face Appointments, it was not an option valued by the younger group.

## Discussion

These results suggest that people with an existing diagnosis of fibromyalgia have distinct preferences for particular features of care. The model of care most preferred is one characterised by diagnosis and ongoing management by a Rheumatologist, via Face-to-face or Phone/video call appointments, with a stronger preference for the latter mode of support. The most preferred treatment was Medication, followed by Physical Therapy, with the least preferred being Talking Therapy. Respondents were indifferent to a Waiting Time for treatment of 6 months but would gain utility from a lower Waiting Time of 3 months and conversely lose utility if they had to wait 12 months. Crucially, respondents appeared willing to receive Ongoing Help and Advice by a Nurse practitioner or a GP, instead of a Specialist Rheumatologist, provided they were compensated by other changes in the model of care. This finding suggests scope to consider different models of skill-mix that would still ensure patients would derive positive levels of utility from the model of care for fibromyalgia.

There are very few previous studies which have considered patients’ preferences for models of care for fibromyalgia. A closely related study [24] was conducted by Valentini et al, where a cross-sectional web survey was undertaken amongst 464 patients who satisfied diagnostic criteria for Fibromyalgia. Respondents were asked to report which treatments they adopted in the past, present and intend to adopt in the future. They found that pharmacological treatment in the past predicted current use of both pharmacological and non-pharmacological treatments, and that use of non-pharmacological treatment in the past was uniquely predictive of its reuse in the present and future. Overall, across the treatments under consideration, pharmacological therapy was most preferred in terms of intention to adopt in the future.

This finding is consistent with our results which showed that pharmacological therapy was the most preferred mode of treatment.

In addition, our findings are also consistent with previous studies that have assessed preferences amongst patient experiencing musculoskeletal conditions using DCEs.

For instance, Klojgaard et al [16] looked at patient preferences for treatment of Low Back Pain (LBP). The DCE’s attributes reflected the treatment, the effects and risks of the treatment and a time component. The main finding was that most patients prefer nonsurgical interventions but, as in our study, they were willing to wait for more ideal outcomes and preferred interventions. The presence of an endowment effect, which suggests patients in general might prefer what they are accustomed to receiving, was also discussed, by which the authors argue that rising surgery rates for LBP might reflect the preferences of doctors and not necessarily patients.

Yi et al [18] investigated the importance of different characteristics of Pain Management Programmes (PMPs) for people with chronic low back pain. They found that method of delivery, travel time and group size were important but providers and contents of PMPs were not the main drivers of preference, unlike our results. Nevertheless, they also found, when taking into account patient heterogeneity, that those with more severe pain preferred PMPs provided by more specialists. It was suggested that, given preferences and resource constraints, more resource intensive PMPs be reserved for those with the most severe and disabling pain.

In terms of strengths and limitations, this was the first DCE exploring the preferences of fibromyalgia patients for an enhanced model of care. It also explored preferences in different population subgroups, with age having been found to have a significant impact. The sample consisted of a large number of fibromyalgia patients. The validity of the DCE was strengthened by deriving attributes and levels from the literature and extensive qualitative research work conducted in other phases of the PACFiND study. The survey was piloted and carefully revised, with a small number of minor changes to question placement and wording being made, according to feedback from respondents. Internal validity was assured by including consistency tests and checking for sensitivity of results through exclusion of respondents who failed them (results are available on request from the authors).

There are limitations associated with the use of a DCE.

First, respondents’ stated preferences are estimated but we cannot be sure respondents would follow through with those preferences in a real-life situation without testing for external validity. Such a test could, in principle, be conducted in a future research study. Second, the attributes were identified based on what the literature and patients suggested but it is possible that important factors exist which were not identified by that exploratory work. For example, communication preferences may be important, with a previous study [25] showing that “effective and open communication” was most important to patients, followed by “patient-centred communication”, with the least important being a “personal communication style”. Related to this point, some of the levels of the attributes were derived from a combination of what is offered in a real setting and what patients would ideally like but that might not be possible within the NHS (e.g., ongoing treatment by a Rheumatologist).

Third, it is possible that patients prefer characteristics of a model of care that are not supported by existing evidence. An example would be patient’s preferences for diagnosis and treatment by a Rheumatologist. Open-text responses revealed that preferences seemed to be based on previous experience of services received. Also, in open-text responses when participants were asked to justify their choices, there was a perception that Rheumatologists provide higher levels of expertise, experience and qualifications; are able to carry out specific tests, therefore ruling out other possible conditions; and the belief that Rheumatologists diagnosis carried more weight with employers and benefits assessors, as well as instilling confidence in the patient. Further, patients who preferred Rheumatologists sometimes described bad experiences with GPs, mainly the view that some GPs still do not believe Fibromyalgia is real. Another example would be the use of Medication as there are no medications specifically licensed for fibromyalgia in the UK and those which are licenced elsewhere or used for associated symptoms have not been rated as useful for patients in terms of effectiveness and safety, e.g. Hauser et al [26]. When asked for their reasoning, patients who chose Medication often felt they alleviated a specific symptom such as pain or lack of sleep. In addition, they tended to have had negative experiences with Talking and Physical Therapy, to the extent that they doubted their effectiveness. Yet however, there appears strong evidence for both therapies, with a meta-analysis from 14 RCTs of telerehabilitation demonstrating statistically significant improvements in fibromyalgia symptoms, pain intensity, pain catastrophising, depression and quality of life [27]. A potential explanation for the disconnect between preference and perceived lack of effect of Talking and Physical Therapy is that, for some patients, Medication was viewed as a precursor to other treatments, chosen because it was felt to have a quicker effect than the other two options.

Finally, the DCE did not explore combinations of treatment (e.g. a preference for both Medication and Physical Therapy vs. Medication alone) or combinations of follow-up (e.g. text messaging and face-to-face appointments vs. text messaging alone), as this would have required a larger sample size.

The study was conducted in the UK and as such patients’ preferences and the characteristics of the model of care are specific to it and might not be generalisable to other countries [26]. For example, the preference for Medication might be reflective of this treatment being the usual choice offered to patients (91% of UK GPs treating patients with fibromyalgia said they used Medications [28], with limited experience of Talking Therapy. This is a common finding and has been described as “status quo bias”, also known as the “endowment effect”, and refers to a situation whereby people value goods more highly once they own them or have experience of them [29,30]. In this case, it might lead respondents to prefer characteristics of the model of care of which they have experience, e.g. Pedley et al [31] looked at the acceptability of a telephone-based cognitive behaviour therapy (tCBT) intervention for individuals with axial SpA, with and without co-morbid Fibromyalgia and found that people’s perceptions of tCBT changed positively over time, even among those who were sceptical at the start). Finally, despite the large size of the sample, it was not designed to be representative of the demographics of UK fibromyalgia patients. Recruitment was conducted via social media and that may have introduced selection bias, excluding patients who are more averse to social media use, although the impact of this bias is difficult to ascertain. Related to this, respondents who choose to take part in all survey research may be more interested in health-related matters and more predisposed to answer in a specific way.

The agreement between the explicitly stated preferences in the questionnaire and the DCE findings across all but one attribute underscores the efficacy of DCE in not only quantifying patients’ preferences but also in revealing preferences that may not be articulated when asked directly. The discrepancy observed in preferences for the mode of Ongoing Help and Advice provision, favouring Phone/video call appointments in the DCE, is likely to reflect a relatively low strength of preference for this particular attribute, with respondents being quite flexible regarding their preferred choice over how ongoing help is delivered, when other features of diagnosis and management are changed.

In conclusion, this study has found that, although respondents prefer Ongoing Help and Advice provided by a specialist, in this case a Rheumatologist, they may be willing to trade-off this preference against other features within a model of care. This preference for follow-up care from a specialist is a common finding when exploring patients’ preferences and the willingness to accept a different skill-mix is particularly important (in this case, appointments with a GP or a Nurse Practitioner), as it may be a more feasible option in settings where the availability of specialist care is highly constrained.

## Supporting information

S1 Fig Example of a choice set. (TIF)

S2 Fig Total WTWD (months) estimates for alternative models of care. (TIF)

S3 Fig WTWD (months) for different age groups. (TIF)

S1 File (DOCX)
