# Supplementary figures and images for: Patient preferences for models of care for fibromyalgia: A discrete choice experiment
Source: PLoS One. 2024 Jun 21;19(6):e0305030. doi: 10.1371/journal.pone.0305030 (PMC11192391; doi:10.1371/journal.pone.0305030)

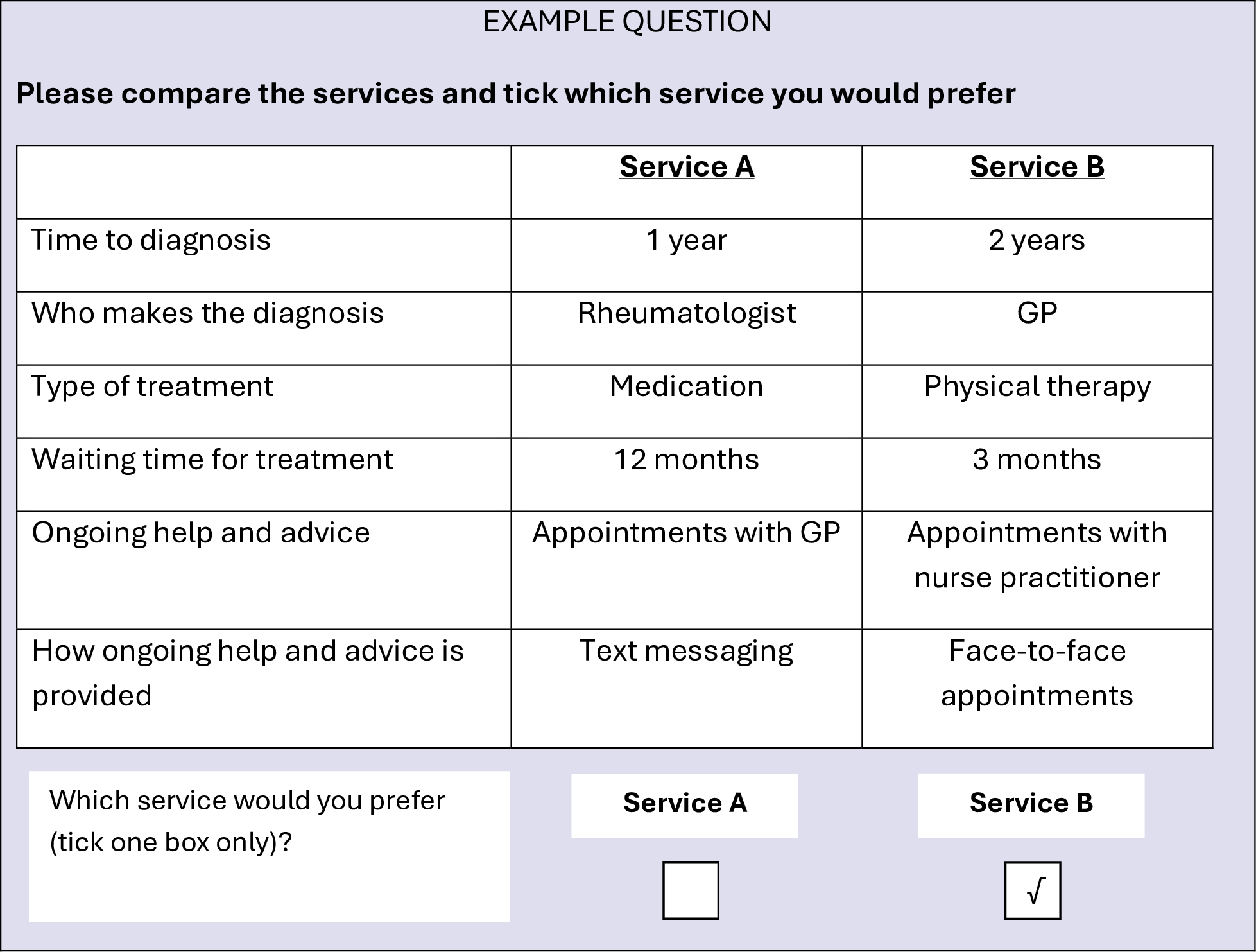

Supplement: S1 Fig — (TIF) [file pone.0305030.s001.tif]

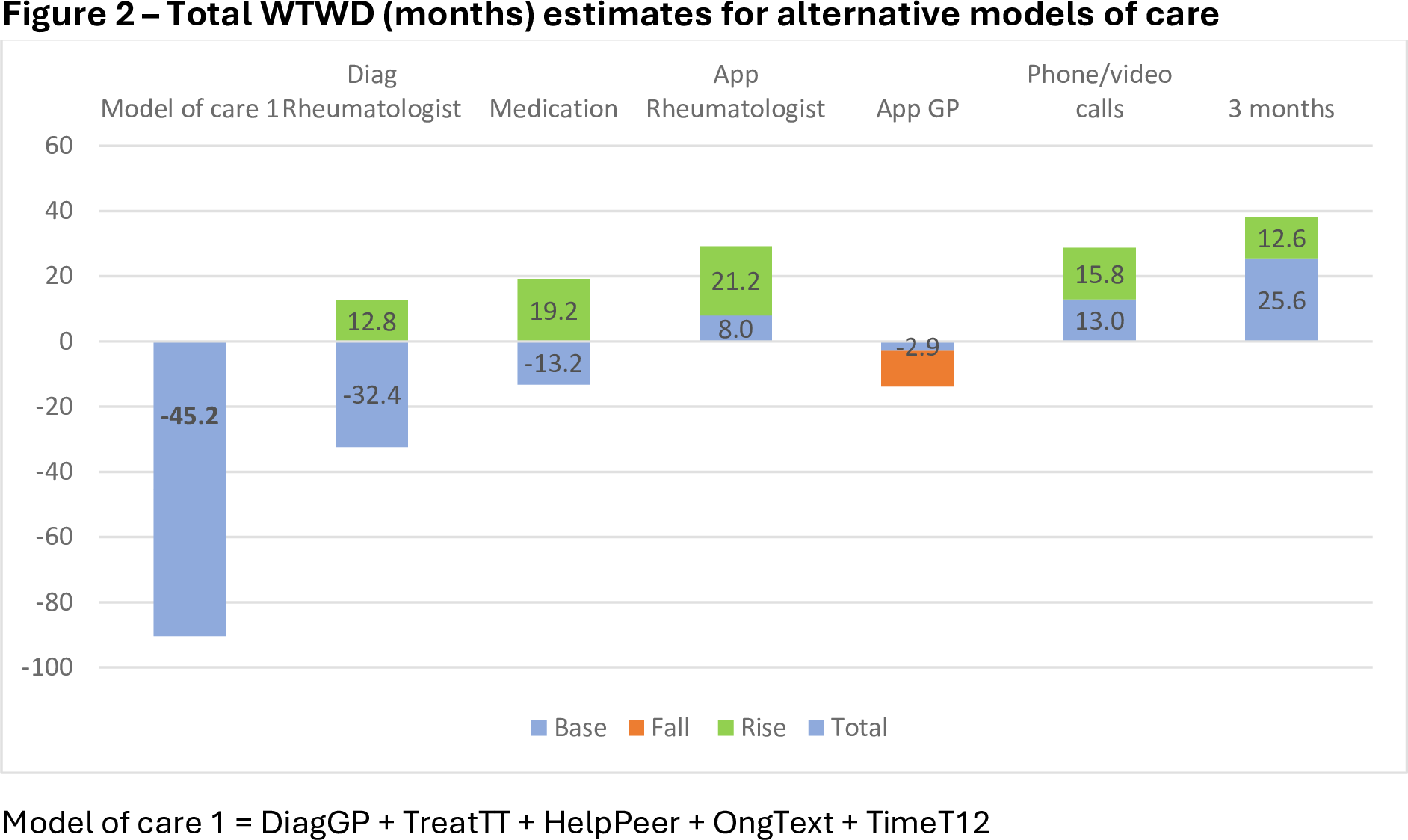

Supplement: S2 Fig — (TIF) [file pone.0305030.s002.tif]

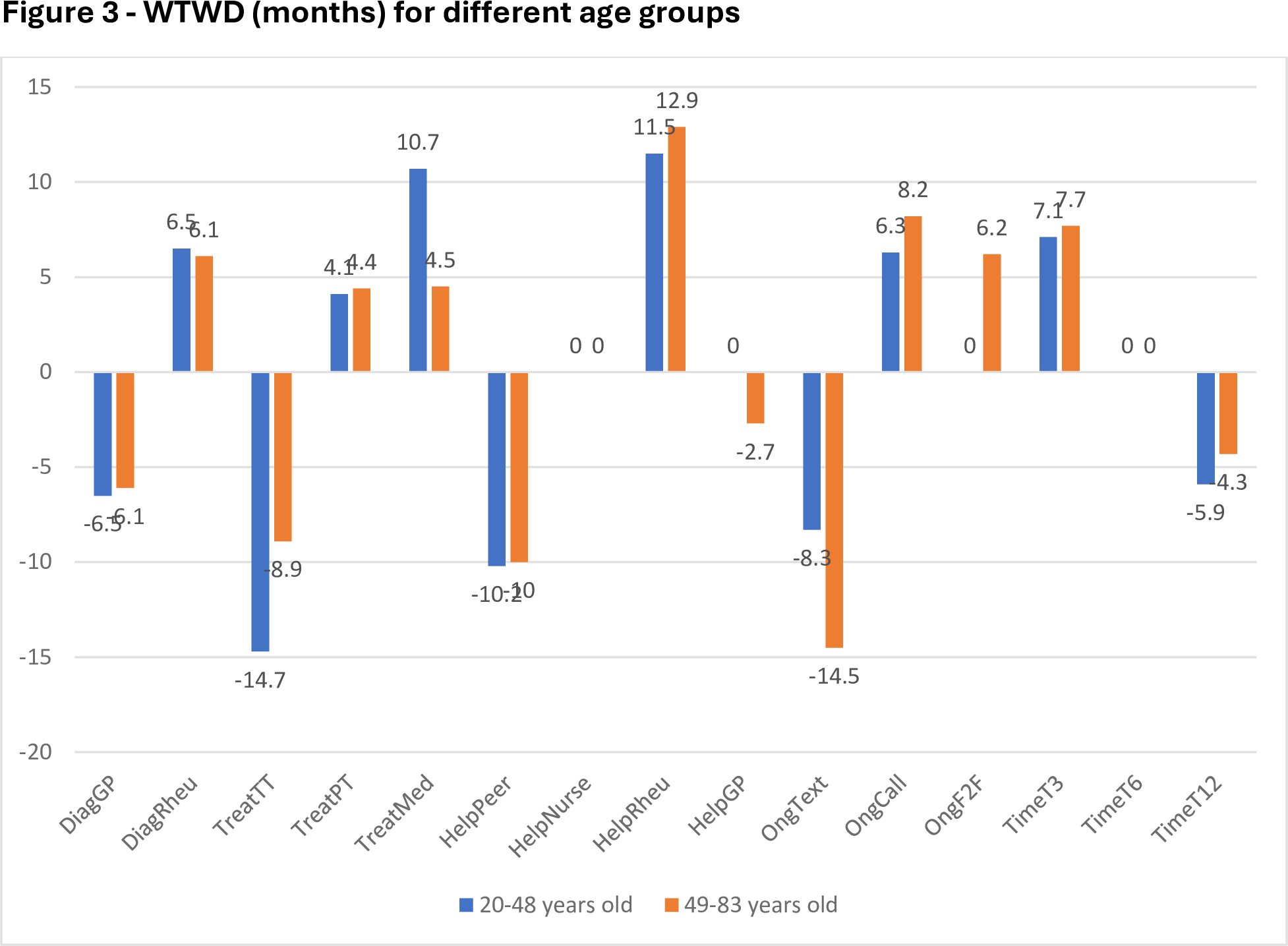

Supplement: S3 Fig — (TIF) [file pone.0305030.s003.tif]
