# Supplementary material for: Patient preferences for models of care for fibromyalgia: A discrete choice experiment
Source: PLoS One. 2024 Jun 21;19(6):e0305030. doi: 10.1371/journal.pone.0305030 (PMC11192391; doi:10.1371/journal.pone.0305030)
Supplement: S1 File — (DOCX) [file pone.0305030.s004.docx]

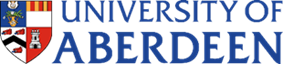


**Developing new models of care for people with fibromyalgia: designing a Discrete Choice Experiment (DCE) to understand patients’ preferences for alternative models of care**

The aim of this survey is to understand patients’ preferences for different types of healthcare for fibromyalgia.

To do this, we would like to understand more about your preferences for healthcare services. We will use the information collected to help develop a new, better model of fibromyalgia care.

If you have a diagnosis of fibromyalgia and live in the UK you are eligible to take part. This study is funded by Versus Arthritis and has been approved by The School Ethics Review Board of the University of Aberdeen (CERB/2021/10/2182).

All information you give as part of this survey is anonymous and confidential. You do not have to take part if you do not wish to do so.

Please answer all the questions. Use the “other” boxes to add any extra information you feel relevant. The survey will take approximately 20 mins to complete.

If you have any questions about this survey you can contact Dr Patricia Norwood by email: [pacfind@abdn.ac.uk](mailto:pacfind@abdn.ac.uk) , or visit our study website: [www.abdn.ac.uk/pacfind](http://www.abdn.ac.uk/pacfind)

**Are you a resident of the UK?**

□ Yes

□ No

**Have you been diagnosed with fibromyalgia?**

□ Yes

□ No

(Only participants who answer yes to both these questions are eligible to take part in the study and will be allowed to complete the survey)

**Informed consent**

·       I confirm that I have read and understand the information above and consent to participate in this survey.

□ Yes, I agree to take part in this study

□ No, I don’t agree to take part in this study

**Section 1 – Introduction**

In this section we want to understand how services for people with fibromyalgia should be organised to better match their needs and preferences.

The main service characteristics that will be considered are:

***Time to diagnosis***

This relates to the time to diagnosis from the start of first symptoms:

- ***1 years***
- ***2 years***
- ***3 years***
- ***5 years***

***Who makes the diagnosis***

This relates to the health care provider that makes the initial diagnosis:

- ***General Practitioner (GP)***
- ***Rheumatologist***

**Type of treatment**

This means the type of help and advice received after the diagnosis. It can be:

- ***Medication –*** You would be prescribed a medicine, or a set of medicines or drugs, used to improve fibromyalgia symptoms.
- ***Physical therapy*** - You would undertake a programme of physical exercise designed by a trained exercise professional.  The type of exercise and the amount of time you do would be adjusted to suit your own needs.
- ***Talking therapy -*** You would undertake a training programme designed by a trained behavioural therapist.  The programme would involve focusing on your current thoughts, beliefs and attitudes, how these affect feelings and behaviour, and learning coping skills to deal with problems.

**Waiting time for treatment**

This refers to, following diagnosis, the waiting time to receive help and advice from a health care provider:

- ***3 months***
- ***6 months***
- ***12 months***

**Ongoing help and advice**

This means ongoing appointments to manage fibromyalgia such as:

- ***Appointments with GP***
- ***Appointments with Rheumatologist***
- ***Appointments with nurse practitioner***
- ***Peer support*** – You would join a group to meet other people with fibromyalgia, to share experiences and learn from each other.

**How ongoing help and advice is provided**

This refers to how the ongoing help and advice would be provided:

- ***Face-to-face appointments***
- ***Phone call/video call appointments***
- ***Text messaging***

In the next section there are 13 questions for you to complete, please answer them all.

**Each question will describe two different services (Service A and Service B) that you could receive. We would like you to tell us which service you would prefer for yourself, if you had a choice.**

- In each question, please imagine that you can only receive one of the two services
- At first glance the questions may appear the same, but each one is different from the next.

When deciding how best to answer the questions, you will need to weigh up different aspects of each service. We understand that some of the choices will be difficult to make, but there are no right or wrong answers. Your personal opinion is what matters.

**Section 2 – What service do you prefer?**

ON THIS PAGE THERE IS AN EXAMPLE OF A CHOICE QUESTION, FOLLOWED BY 13 QUESTIONS FOR YOU TO ANSWER.

PLEASE READ THE EXAMPLE BELOW BEFORE COMPLETING THE REST OF THE QUESTIONNAIRE.

**EXAMPLE CHOICE QUESTION**

| EXAMPLE QUESTION  **Please compare the services and tick which service you would prefer**   \|  \| **Service A** \| **Service B** \| \| --- \| --- \| --- \| \| Time to diagnosis \| 1 year \| 2 years \| \| Who makes the diagnosis \| Rheumatologist \| GP \| \| Type of treatment \| Medication \| Physical therapy \| \| Waiting time for treatment \| 12 months \| 3 months \| \| Ongoing help and advice \| Appointments with GP \| Appointments with nurse practitioner \| \| How ongoing help and advice is provided \| Text messaging \| Face-to-face appointments \|     **Service A**  **Service B**  Which service would you prefer (tick one box only)?  √ |
| --- | --- | --- | --- | --- | --- | --- | --- | --- | --- | --- | --- | --- | --- | --- | --- | --- | --- | --- | --- | --- | --- |

By choosing **Service B**, this person would take **2 years** to get a diagnosis from a **GP**. Treatment would consist of **physical therapy** and it would start **3 months** after diagnosis. The ongoing help and advice would be provided by a **nurse practitioner** in **face-to-face appointments**.

| **QUESTION 1**  **Please compare the services and tick which service you would prefer**   \|  \| **Service A** \| **Service B** \| \| --- \| --- \| --- \| \| Time to diagnosis \| 5 years \| 1 year \| \| Who makes the diagnosis \| Rheumatologist \| GP \| \| Type of treatment \| Medication \| Physical Therapy \| \| Waiting time for treatment \| 12 months \| 3 months \| \| Ongoing help and advice \| Peer support \| Appointments with GP \| \| How ongoing help and advice is provided \| Phone call/video call appointments \| Face-to-face appointments \|     **Service A**  **Service B**  Which service would you prefer (tick one box only)? |
| --- | --- | --- | --- | --- | --- | --- | --- | --- | --- | --- | --- | --- | --- | --- | --- | --- | --- | --- | --- | --- | --- |
| **QUESTION 2**  **Please compare the services and tick which service you would prefer**   \|  \| **Service A** \| **Service B** \| \| --- \| --- \| --- \| \| Time to diagnosis \| 1 year \| 5 years \| \| Who makes the diagnosis \| GP \| Rheumatologist \| \| Type of treatment \| Talking Therapy \| Medication \| \| Waiting time for treatment \| 6 months \| 6 months \| \| Ongoing help and advice \| Appointments with nurse practitioner \| Appointments with GP \| \| How ongoing help and advice is provided \| Text messaging \| Face-to-face appointments \|     **Service A**  **Service B**  Which service would you prefer (tick one box only)? |

| **QUESTION 3**  **Please compare the services and tick which service you would prefer**   \|  \| **Service A** \| **Service B** \| \| --- \| --- \| --- \| \| Time to diagnosis \| 1 year \| 5 years \| \| Who makes the diagnosis \| Rheumatologist \| GP \| \| Type of treatment \| Physical Therapy \| Talking Therapy \| \| Waiting time for treatment \| 12 months \| 3 months \| \| Ongoing help and advice \| Appointments with nurse practitioner \| Appointments with Rheumatologist \| \| How ongoing help and advice is provided \| Face-to-face appointments \| Text messaging \|     **Service A**  **Service B**  Which service would you prefer (tick one box only)? |
| --- | --- | --- | --- | --- | --- | --- | --- | --- | --- | --- | --- | --- | --- | --- | --- | --- | --- | --- | --- | --- | --- |
| **QUESTION 4**  **Please compare the services and tick which service you would prefer**   \|  \| **Service A** \| **Service B** \| \| --- \| --- \| --- \| \| Time to diagnosis \| 1 year \| 5 years \| \| Who makes the diagnosis \| Rheumatologist \| GP \| \| Type of treatment \| Medication \| Physical Therapy \| \| Waiting time for treatment \| 6 months \| 6 months \| \| Ongoing help and advice \| Peer support \| Appointments with nurse practitioner \| \| How ongoing help and advice is provided \| Text messaging \| Phone call/video call appointments \|     **Service A**  **Service B**  Which service would you prefer (tick one box only)? |
| **QUESTION 5**  **Please compare the services and tick which service you would prefer**   \|  \| **Service A** \| **Service B** \| \| --- \| --- \| --- \| \| Time to diagnosis \| 3 years \| 3 years \| \| Who makes the diagnosis \| GP \| Rheumatologist \| \| Type of treatment \| Medication \| Talking Therapy \| \| Waiting time for treatment \| 12 months \| 3 months \| \| Ongoing help and advice \| Appointments with GP \| Peer support \| \| How ongoing help and advice is provided \| Text messaging \| Face-to-face appointments \|     **Service A**  **Service B**  Which service would you prefer (tick one box only)? |
| **QUESTION 6**  **Please compare the services and tick which service you would prefer**   \|  \| **Service A** \| **Service B** \| \| --- \| --- \| --- \| \| Time to diagnosis \| 3 years \| 1 year \| \| Who makes the diagnosis \| GP \| Rheumatologist \| \| Type of treatment \| Talking Therapy \| Physical Therapy \| \| Waiting time for treatment \| 6 months \| 12 months \| \| Ongoing help and advice \| Appointments with GP \| Appointments with Rheumatologist \| \| How ongoing help and advice is provided \| Phone call/video call appointments \| Text messaging \|     **Service A**  **Service B**  Which service would you prefer (tick one box only)? |

| **QUESTION 7**  **Please compare the services and tick which service you would prefer**   \|  \| **Service A** \| **Service B** \| \| --- \| --- \| --- \| \| Time to diagnosis \| 2 years \| 2 years \| \| Who makes the diagnosis \| Rheumatologist \| GP \| \| Type of treatment \| Physical Therapy \| Medication \| \| Waiting time for treatment \| 3 months \| 12 months \| \| Ongoing help and advice \| Appointments with GP \| Appointments with nurse practitioner \| \| How ongoing help and advice is provided \| Phone call/video call appointments \| Face-to-face appointments \|     **Service A**  **Service B**  Which service would you prefer (tick one box only)? |
| --- | --- | --- | --- | --- | --- | --- | --- | --- | --- | --- | --- | --- | --- | --- | --- | --- | --- | --- | --- | --- | --- |
| **QUESTION 8**  **Please compare the services and tick which service you would prefer**   \|  \| **Service A** \| **Service B** \| \| --- \| --- \| --- \| \| Time to diagnosis \| 3 years \| 2 years \| \| Who makes the diagnosis \| Rheumatologist \| GP \| \| Type of treatment \| Talking Therapy \| Physical Therapy \| \| Waiting time for treatment \| 3 months \| 6 months \| \| Ongoing help and advice \| Appointments with nurse practitioner \| Peer support \| \| How ongoing help and advice is provided \| Face-to-face appointments \| Phone call/video call appointments \|     **Service A**  **Service B**  Which service would you prefer (tick one box only)? |
| **QUESTION 9**  **Please compare the services and tick which service you would prefer**   \|  \| **Service A** \| **Service B** \| \| --- \| --- \| --- \| \| Time to diagnosis \| 2 years \| 3 years \| \| Who makes the diagnosis \| GP \| Rheumatologist \| \| Type of treatment \| Talking Therapy \| Medication \| \| Waiting time for treatment \| 12 months \| 3 months \| \| Ongoing help and advice \| Appointments with Rheumatologist \| Appointments with nurse practitioner \| \| How ongoing help and advice is provided \| Face-to-face appointments \| Text Messaging \|     **Service A**  **Service B**  Which service would you prefer (tick one box only)? |
| **QUESTION 10**  **Please compare the services and tick which service you would prefer**   \|  \| **Service A** \| **Service B** \| \| --- \| --- \| --- \| \| Time to diagnosis \| 2 years \| 3 years \| \| Who makes the diagnosis \| GP \| Rheumatologist \| \| Type of treatment \| Medication \| Talking Therapy \| \| Waiting time for treatment \| 3 months \| 12 months \| \| Ongoing help and advice \| Appointments with Rheumatologist \| Appointments with GP \| \| How ongoing help and advice is provided \| Phone call/video call appointments \| Text messaging \|     **Service A**  **Service B**  Which service would you prefer (tick one box only)? |
| **QUESTION 11**  **Please compare the services and tick which service you would prefer**   \|  \| **Service A** \| **Service B** \| \| --- \| --- \| --- \| \| Time to diagnosis \| 5 years \| 1 year \| \| Who makes the diagnosis \| Rheumatologist \| GP \| \| Type of treatment \| Physical Therapy \| Medication \| \| Waiting time for treatment \| 6 months \| 6 months \| \| Ongoing help and advice \| Appointments with Rheumatologist \| Peer support \| \| How ongoing help and advice is provided \| Text messaging \| Phone call/video call appointments \|     **Service A**  **Service B**  Which service would you prefer (tick one box only)? |
| **QUESTION 12**  **Please compare the services and tick which service you would prefer**   \|  \| **Service A** \| **Service B** \| \| --- \| --- \| --- \| \| Time to diagnosis \| 5 years \| 2 years \| \| Who makes the diagnosis \| GP \| Rheumatologist \| \| Type of treatment \| Physical Therapy \| Talking Therapy \| \| Waiting time for treatment \| 3 months \| 12 months \| \| Ongoing help and advice \| Peer support \| Appointments with Rheumatologist \| \| How ongoing help and advice is provided \| Face-to-face appointments \| Phone call/video call appointments \|     **Service A**  **Service B**  Which service would you prefer (tick one box only)? |
| **QUESTION 13**  **Please compare the services and tick which service you would prefer**   \|  \| **Service A** \| **Service B** \| \| --- \| --- \| --- \| \| Time to diagnosis \| 3 years \| 1 year \| \| Who makes the diagnosis \| GP \| Rheumatologist \| \| Type of treatment \| Talking Therapy \| Physical Therapy \| \| Waiting time for treatment \| 6 months \| 12 months \| \| Ongoing help and advice \| Appointments with GP \| Appointments with Rheumatologist \| \| How ongoing help and advice is provided \| Phone call/video call appointments \| Text messaging \|     **Service A**  **Service B**  Which service would you prefer (tick one box only)? |

14. Please now rank all of the attributes you have seen in the previous questions in order of importance (enter “1” next to the most important, “2” for next most important, etc., and “6” for least important.

| Time to diagnosis |  |
| --- | --- |
| Who makes the diagnosis |  |
| Type of treatment |  |
| Waiting time for treatment |  |
| Ongoing help and advice |  |
| How ongoing help and advice is provided |  |

15. If you could have chosen the health care provider to make your diagnosis, who would that have been?

- GP

- Rheumatologist

15.1. Why would you have preferred that health care provider?

16. If you could have chosen the type of help and advice provided after you were diagnosed, which one would you have preferred? Please chose one option only.

- Medication
- Physical Therapy
- Talking Therapy

16.1. Why would you have preferred that type of treatment?

17. If you could have chosen the type of ongoing help and advice provided, which one would you have chosen? Please chose one option only.

- Appointments with GP
- Appointments with Rheumatologist
- Appointments with nurse practitioner
- Peer support

17.1. Why would you have chosen that type of ongoing help and advice?

18. If you could have chosen how ongoing help and advice is provided, which one of these options would you have preferred? Please chose one option only.

- Face-to-face appointments

- Phone call/video call appointments

- Text messaging

18.1. Why would you have preferred that option?

**Section 3 – About you**

We would like to ask a few questions about you, so that we may better understand your answers to the questionnaire. We would like to remind you that **all** information collected is **confidential and anonymous.**

19. What is your age (years)?

20. What is your gender?

1. Male
2. Female
3. Non-binary/Third gender
4. Prefer not to say

21. What year were you diagnosed with fibromyalgia?

22. Please select the category that best describes your current employment status:

1. Part-time Employment
2. Full-time Employment
3. Unemployed
4. Retired
5. Student
6. Other (please specify) _________________

23. Please circle highest level of formal education you have completed:

1) Secondary School

2) Vocational/Trade/College Qualification

3) Highers / A Levels

4) University Qualification

24. Please select the category that represents your household income from all sources (before tax and other deductions):

1) Up to £10,000

2) £10,001 - £20,000

3) £20,001 - £30,000

4) £30,001 - £40,000

5) £40,001 - £50,000

6) £50,001 +

25. Do you have any comments about this questionnaire?

26. Where did you hear about this survey?
